# Quantitative Ultrasound-Based Precision Diagnosis of Papillary, Follicular, and Medullary Thyroid Carcinomas Using Morphological, Structural, and Textural Features

**DOI:** 10.3390/cancers17172761

**Published:** 2025-08-24

**Authors:** Hanna Piotrzkowska Wróblewska, Piotr Karwat, Agnieszka Żyłka, Katarzyna Dobruch Sobczak, Marek Dedecjus, Jerzy Litniewski

**Affiliations:** 1 Ultrasound Department, Institute of Fundamental Technological Research, Polish Academy of Sciences, 02-106 Warsaw, Poland; 2 Department of Endocrine Oncology and Nuclear Medicine, Maria Sklodowska-Curie National Research Institute of Oncology, 02-781 Warsaw, Poland; 3 Radiology Department II, Maria Sklodowska-Curie National Research Institute of Oncology, 02-034 Warsaw, Poland; kdsobczak@gmail.com

**Keywords:** thyroid cancer, ultrasound imaging, quantitative analysis, machine learning, papillary thyroid carcinoma, follicular thyroid carcinoma, medullary thyroid carcinoma

## Abstract

**Background/Objectives**: Thyroid cancer encompasses distinct histological subtypes with varying biological behavior and treatment implications. Accurate preoperative subtype differentiation remains challenging. Although ultrasound (US) is widely used for thyroid nodule evaluation, qualitative assessment alone is often insufficient to distinguish between papillary (PTC), follicular (FTC), and medullary thyroid carcinoma (MTC). **Methods:** A retrospective analysis was performed on patients with histologically confirmed PTC, FTC, or MTC. A total of 224 standardized B-mode ultrasound images were analyzed. A set of fully quantitative features was extracted, including morphological characteristics (aspect ratio and perimeter-to-area ratio), internal echotexture (echogenicity and local entropy), boundary sharpness (gradient measures and KL divergence), and structural components (calcifications and cystic areas). Feature extraction was conducted using semi-automatic algorithms implemented in MATLAB. Statistical differences were assessed using the Kruskal–Wallis and Dunn–Šidák tests. A Random Forest classifier was trained and evaluated to determine the discriminatory performance of individual and combined features. **Results:** Significant differences (*p* < 0.05) were found among subtypes for key features such as perimeter-to-area ratio, normalized echogenicity, and calcification pattern. The full-feature Random Forest model achieved an overall classification accuracy of 89.3%, with F1-scores of 93.4% for PTC, 85.7% for MTC, and 69.1% for FTC. A reduced model using the top 10 features yielded an even higher accuracy of 91.8%, confirming the robustness and clinical relevance of the selected parameters. **Conclusions:** Subtype classification of thyroid cancer was effectively performed using quantitative ultrasound features and machine learning. The results suggest that biologically interpretable image-derived metrics may assist in preoperative decision-making and potentially reduce the reliance on invasive diagnostic procedures.

## Simple Summary

Thyroid cancer includes several types that differ in how they grow and how they should be treated. Although ultrasound is widely used to examine thyroid nodules, it can be difficult to determine which type of cancer is present using standard imaging alone. In this study, we applied a computer-based method to automatically measure and analyze ultrasound features of thyroid tumors. By using machine learning techniques, we distinguished between three common types of thyroid cancer: papillary, follicular, and medullary. We found that certain features, such as tumor shape, brightness, and internal structure, were helpful in identifying the cancer subtype. This approach could support doctors in making more accurate diagnoses, reduce unnecessary procedures such as biopsies, and guide more personalized treatment decisions.

## 1. Introduction

Thyroid cancer (TC) is the most common malignancy of the endocrine system, and its global incidence has steadily increased in recent decades. According to GLOBOCAN 2020 data, more than 586,000 new TC cases are diagnosed each year, with a strong predominance in women [1]. In Poland alone, over 4000 cases were reported in 2021, confirming the significance of this disease at the population level [2]. Although this trend is partly due to improved access to high-resolution ultrasound (US) and widespread screening, a true increase in incidence is also suspected [3,4].

Thyroid malignancies encompass diverse histopathological subtypes with distinct biological behavior, prognoses, and treatment strategies. Papillary thyroid carcinoma (PTC) is the most common subtype (80–85%) and is generally associated with excellent prognosis and indolent progression [4,5,6]. Follicular thyroid carcinoma (FTC), comprising 10–15% of cases, may follow a more aggressive course, especially in the presence of vascular invasion or distant metastases [4,5,6]. Medullary thyroid carcinoma (MTC), arising from parafollicular C cells, accounts for 1–2% of thyroid cancers and often presents as part of inherited syndromes such as MEN 2A and 2B. Due to its neuroendocrine origin, MTC requires additional biochemical testing (e.g., serum calcitonin), genetic screening, and a different surgical approach [6,7,8].

Accurate preoperative differentiation between these subtypes—particularly between PTC, FTC, and MTC—is essential for treatment planning. Ultrasound remains the primary diagnostic tool for evaluating thyroid nodules, as it is safe, widely accessible, and highly sensitive for solid lesions. Multiparametric ultrasound further enables detailed assessment of lesion morphology, facilitates initial risk stratification, and supports biopsy qualification. Various risk stratification systems, including ACR TI-RADS, EU-TIRADS, and EU-TIRADS-PL, have been developed to standardize nodule descriptions and reduce unnecessary fine-needle aspiration biopsies (FNABs) [9,10,11]. However, these systems only assess malignancy risk without providing insight into the histopathological subtype.

The ability to distinguish PTC, FTC, and MTC based on sonographic features may provide critical support in clinical decision-making [12,13,14]. Although several studies have described typical ultrasound features of individual subtypes, comprehensive comparative analyses directly contrasting PTC, FTC, and MTC remain scarce [15,16,17,18,19,20,21,22,23]. Table 1 summarizes the most frequently reported ultrasound features for these three main subtypes, highlighting differences in echogenicity, margins, calcifications, vascularity, and stiffness [12,13,14,15,16,17,18,19,20,21,22,23].

Medullary thyroid carcinoma (MTC) can often mimic benign lesions on ultrasound. It typically presents as a larger, solid, hypoechoic nodule with smooth margins, rich internal vascularity, and coarse (macro-) calcifications. Compared to papillary thyroid carcinoma (PTC), MTC less frequently displays irregular margins or microcalcifications and is more likely to exhibit increased intranodular blood flow. The ultrasound appearance of MTC can be difficult to distinguish from that of benign nodules, particularly when smooth margins are present [13,24,25].

In contrast, PTC is more commonly associated with features suggestive of malignancy, such as irregular margins, microcalcifications, hypoechogenicity, and a “taller-than-wide” shape. Classic PTC typically demonstrates more ultrasound features of malignancy compared to the follicular variant (FVPTC), which more frequently shows smooth margins and fewer microcalcifications. Consequently, FVPTC may be more challenging to identify based solely on ultrasound appearance, as it often lacks the typical high-risk features [22,26,27].

Despite advances in ultrasound imaging techniques, differentiating thyroid cancer subtypes based solely on B-mode imaging remains a diagnostic challenge due to considerable overlap in sonographic features. In recent years, there has been growing interest in the application of quantitative image analysis and machine learning algorithms in the evaluation of thyroid nodules [28,29]. However, the majority of existing studies have focused on binary classification (benign vs. malignant), without accounting for histological differentiation.

The aim of this study was to evaluate whether automatically extracted multiparametric ultrasound features can be used to distinguish between the three major histological subtypes of thyroid cancer: papillary (PTC), follicular (FTC), and medullary (MTC). The objective was to assess the diagnostic value of quantitative imaging parameters in supporting accurate subtype classification and informing personalized therapeutic strategies.

The novelty of our work lies in moving beyond the binary paradigm of thyroid nodule assessment. While most prior ultrasound studies have focused on distinguishing benign from malignant lesions, direct quantitative comparisons among papillary, follicular, and medullary carcinomas remain scarce. In contrast to qualitative TI-RADS descriptors, which are subjective and prone to interobserver variability, we employed fully quantitative and standardized imaging features that are clinically meaningful. Our approach integrates multiple domains of information—morphological shape metrics, echogenicity, margin sharpness, calcification distribution, and textural parameters—into a unified classification model. Compared with previous ultrasound-based studies relying mainly on qualitative descriptors or handcrafted single-domain features [15,16,17,18,19,20,21,22,23], our method leverages a systematic, multiparametric, and automated analysis pipeline. Furthermore, unlike CT or MRI, which provide complementary but less accessible diagnostic information, B-mode ultrasound is widely available, cost-effective, and safe. By enhancing its diagnostic capability through quantitative analysis, our method aims to provide a practical and transparent tool for improving preoperative subtype differentiation.

## 2. Materials and Methods

### 2.1. Study Design and Patient Cohort

The study included patients who underwent surgery between 2021 and 2022 at the Department of Oncological Endocrinology and Nuclear Medicine, Maria Sklodowska-Curie National Research Institute of Oncology in Warsaw. The initial dataset comprised 214 thyroid nodules that were evaluated by ultrasound and subjected to ultrasound-guided fine-needle aspiration biopsy (FNAB). This cohort included benign lesions, borderline tumors, and nodules with malignant potential. For the purpose of this study, only cases with postoperative histopathological confirmation of one of the three main malignant subtypes—papillary thyroid carcinoma (PTC, n = 90), follicular thyroid carcinoma (FTC, n = 14), or medullary thyroid carcinoma (MTC, n = 18)—were included, while benign nodules, borderline tumors, and anaplastic thyroid carcinomas were excluded. In patients with multiple nodules, the dominant lesion—defined as the largest or most suspicious on ultrasound—was selected for analysis. The final study group consisted of 122 patients (104 women and 18 men).

Clinical information was collected for all cases. The mean age of the patients was 48.3 years (range: 22–85). Lesions were located in the right lobe in 62 cases, the left lobe in 53 cases, and the isthmus in 7 cases. Nodule size was measured in three orthogonal dimensions (anteroposterior, transverse, and longitudinal), with the maximum diameter used for descriptive statistics (mean: 19.1 mm, range: 4–92 mm). According to the EU-TIRADS classification, 9 nodules were categorized as EU-TIRADS 3, 16 as EU-TIRADS 4, and 97 as EU-TIRADS 5. Cytological findings were classified according to the Bethesda System: 4 nodules were category II, 4 were category III (AUS/FLUS), 19 were category IV, 57 were category V, and 38 were category VI.

Pathological staging was determined based on the AJCC/UICC TNM classification. The majority of lesions were staged as pT1aN0 (n = 47) and pT1bN0 (n = 21), with smaller groups corresponding to pT1aNx (n = 4), pT1aN1a (n = 2), pT1aN1b (n = 1), pT1bNx (n = 7), pT1bN1a (n = 2), and pT1bN1b (n = 6). For T2 tumors, staging included pT2Nx (n = 5), pT2N0 (n = 14), pT2N1a (n = 1), and pT2N1b (n = 1). Less frequent were T3 lesions: pT3aNx (n = 1), pT3aN0 (n = 5), and pT3aN1b (n = 4). Only one case was classified as pT4aN1b, while no tumors were staged as pT3b or pT4b.

Surgical treatment included either total thyroidectomy or lobectomy with isthmectomy, and in many cases it was complemented by central neck compartment lymphadenectomy. The extent of surgery was determined individually by the surgical oncologist, based on clinical presentation, ultrasound findings, and cytological evaluation.

Ultrasound images were anonymized, and analyses were conducted retrospectively using archived data. The structure of the study cohort reflected the known epidemiological predominance of PTC over other thyroid cancer subtypes [30,31,32,33].

The study was approved by the Bioethics Committee of the Maria Sklodowska-Curie National Research Institute of Oncology in Warsaw (approval number 83/2021). Written informed consent was obtained from all participants prior to inclusion in the study.

### 2.2. Image Acquisition and Preprocessing

B-mode ultrasound images of focal thyroid lesions were acquired from 122 patients with histopathologically confirmed malignant tumors. For each case, two orthogonal images—transverse and longitudinal—were obtained, resulting in a total of 244 images for further analysis.

All examinations were performed using a Philips Epiq 5 ultrasound system equipped with a high-frequency linear transducer (eL18-4, 4–18 MHz). Images were acquired in B-mode at a central frequency of approximately 12–18 MHz, with depth settings ranging from 3.0 to 4.2 cm to fully visualize the thyroid gland and the focal lesion. The dynamic range was set to 68 dB, and overall gain was adjusted individually for each patient within the range of 40–55% to optimize image contrast. A single focal zone was positioned at the center of the lesion and, in some cases, at the lower margin of the lesion to optimize boundary visualization. Both transverse and longitudinal planes were acquired for each nodule.

Subsequent image processing was performed in the MATLAB environment (MathWorks, Natick, MA, USA). Binary masks of each lesion were generated, initially created manually by physicians and then refined semi-automatically using morphological operations and an active contour algorithm. The agreement between the initial and final mask was assessed using the Dice similarity coefficient (0.9637 ± 0.013), which in this context reflected the extent of correction introduced by the algorithm relative to the manual segmentation. It should be noted that, in our study, the Dice coefficient was not used to compare the result to an independent reference mask, but rather to quantify the degree of modification introduced by the algorithm relative to the initial segmentation. The spatial resolution of the images was determined based on metadata from the ultrasound device, enabling the conversion of all pixel-based measurements into millimeter (mm) units. This allowed for reliable extraction of geometric and structural features of the analyzed lesions.

All ultrasound images and their corresponding segmentation masks were anonymized.

### 2.3. Quantitative Feature Extraction

#### 2.3.1. Morphological Features

- Aspect Ratio (Height-to-Width Ratio)

One of the fundamental and most commonly used morphological features of focal thyroid lesions in clinical practice is their size and shape, including the height-to-width ratio, known as the aspect ratio. For each lesion, a minimum bounding rectangle was defined in the transverse plane, in accordance with the recommendations of the American Thyroid Association and the EU-TIRADS and ACR-TIRADS systems [4,9,10,11].

Mathematically, the aspect ratio was defined as follows:

(1) $$AR\;=\;\frac{H}{W}$$

where H denotes the anteroposterior (height) dimension of the lesion and W the transverse (width) dimension. A value greater than 1 (so-called “taller-than-wide”) indicates predominant growth along the anteroposterior axis, which is typical for infiltrative lesions and is associated with a higher risk of malignancy [34,35,36,37,38].

In the context of thyroid cancer subtype differentiation, this feature is particularly characteristic of papillary thyroid carcinoma (PTC), which more frequently exhibits a “taller-than-wide” shape compared to follicular (FTC) or medullary thyroid carcinoma (MTC). In contrast, FTC and MTC more often present with regular proportions, which may lead to misclassification as benign lesions [14,36,39,40,41].

- Shape Complexity (Perimeter-to-Area Ratio)

To obtain accurate binary masks of focal thyroid lesions, a semi-automatic refinement method was applied, based on initial manual segmentations performed by experienced clinicians. The segmentation process began with morphological opening to eliminate small artifacts while preserving the overall integrity of the lesion shape. Subsequently, the mask contour was refined using an active contour model, which minimized the total contour energy by incorporating an internal term (promoting contour smoothness) and an external term (attracting the contour toward lesion boundaries based on image intensity gradients). The active contour algorithm was iterated up to 200 times or until convergence of the contour was achieved.

Based on the final masks, the shape complexity of each lesion was quantified using the perimeter-to-area ratio (PAR). This feature was defined as follows:

(2) $$PAR=\frac{P}{A}$$

where P represents the length of the lesion boundary (perimeter) and A the enclosed lesion area. The perimeter was obtained from the binary mask contour length, while the area was calculated as the number of pixels within the mask, converted to mm^2^ according to the pixel spacing in both imaging axes.

Higher PAR values reflect increased boundary irregularity, which may indicate invasive growth. This parameter is particularly useful for differentiating thyroid cancer subtypes, especially in identifying lesions with irregular, spiculated borders typical of PTC. In contrast, FTC and MTC more frequently exhibit smooth, well-defined borders [42,43,44].

#### 2.3.2. Echogenicity and Internal Echotexture Features

- Echogenicity

To quantitatively assess the echogenicity of each focal thyroid lesion, RGB ultrasound images were converted to single-channel grayscale format and normalized to a [0, 1] intensity range. Using the corresponding binary segmentation mask, only the pixels within the lesion area were extracted, excluding the background and adjacent anatomical structures.

To minimize the influence of artifacts and technical inhomogeneities, pixels with extreme brightness values—above 0.85 (which may correspond to calcifications) and below 0.2 (potentially representing cystic or fluid-filled regions)—were excluded from further analysis.

For each lesion, the mean ($\mu_{lesion}$) and median ($M_{lesion}$) grayscale intensities were computed within the lesion mask. To account for inter-patient variability and reduce dependence on ultrasound system settings, these values were normalized to the mean intensity of the surrounding normal thyroid parenchyma ($\mu_{thyroid}$):

(3) $${NE}_{mean}=\frac{\mu_{lesion}\;}{\mu_{thyroid}},\;{NE}_{median}=\frac{M_{lesion}\;}{\mu_{thyroid}}$$

where ${NE}_{mean}$ and ${NE}_{median}$ denote the normalized mean and median echogenicity, respectively.

Normalization provided a patient-specific reference, ensuring that relative rather than absolute echogenicity values were compared across cases. This step minimized bias from gain settings, probe type, or depth-related attenuation, making the echogenicity features more robust and reproducible across the study cohort.

- Internal Echotexture Features

To quantitatively assess the internal echotexture of each lesion, both global and local intensity variability were analyzed.

Global heterogeneity was estimated by computing the standard deviation of grayscale intensity values within the binary lesion mask:

(4) $$\sigma =\sqrt{\frac{1}{N}\sum_{i=1}^{N}{\left(I_{i}-\mu \right)}^{2}}$$

where $I_{i}$ denotes the intensity of the *i*-th pixel inside the lesion mask, μ is the mean intensity of the lesion, and N is the total number of pixels in the lesion.

Local disorder was assessed using entropy. For each pixel, entropy was computed in a circular neighborhood of radius 0.7 mm:

(5) $$E=-\sum_{k=1}^{L}p_{k}log(p_{k})$$

where $p_{k}$ denotes the probability of intensity level *k* within the local neighborhood and *L* is the number of quantized intensity levels. The mean entropy value across all pixels within the lesion was then used as a marker of microstructural disorganization.

In addition to these intensity-based descriptors, second-order texture features were extracted from the gray-level co-occurrence matrix (GLCM). Given a displacement vector (Δ*x*,Δ*y*), the normalized GLCM P(i,j) encodes the probability of finding a pair of gray levels, *i* and *j*, at that spatial offset. From this matrix, four features were computed:

(6) $$Contrast=\sum_{i=1}^{L}\sum_{j=1}^{L}{\left(i-j\right)}^{2}P(i,j)$$

measuring local brightness variations and sharp transitions,

(7) $$Correlation=\sum_{i=1}^{L}\sum_{j=1}^{L}\frac{\left(i-\mu_{i}\right)\left(j-\mu_{j}\right)P\left(i,j\right)}{\sigma_{i}\sigma_{j}}$$

capturing linear dependencies between neighboring intensities, where $\mu_{i}$, $\mu_{j}$, $\sigma_{i}$, and $\sigma_{j}$ are marginal means and standard deviations of the distribution,

(8) $$Homogenity=\sum_{i=1}^{L}\sum_{j=1}^{L}\frac{P\left(i,j\right)}{1+\left|i-j\right|}$$

reflecting local uniformity, with higher values for smoother textures,

(9) $$Energy=\sum_{i=1}^{L}\sum_{j=1}^{L}P{\left(i,j\right)}^{2}$$

quantifying the degree of repetition in brightness patterns.

Higher values of standard deviation and entropy indicate increased echotextural inhomogeneity [45], whereas the GLCM-derived features capture more subtle patterns of pixel interrelationships. These quantitative descriptors may reveal microstructural differences among thyroid cancer subtypes, potentially supporting improved diagnostic discrimination [46,47,48,49].

#### 2.3.3. Boundary Characteristics

- Assessment of Lesion Boundary Sharpness

To quantitatively evaluate the sharpness of lesion boundaries on B-mode ultrasound images, two complementary approaches were applied: (1) analysis of intensity gradients along the lesion contour and (2) evaluation of local intensity transitions across the lesion margin.

All grayscale images were first normalized relative to the mean echogenicity of adjacent normal thyroid parenchyma. For each point along the lesion contour, a margin region with a total width of 1 mm (±0.5 mm on each side) was defined, incorporating pixels both inside and outside the lesion. Descriptive statistics of these intensity changes—means, medians, and standard deviations—were computed to characterize the overall contrast along the boundary.

In addition, for each contour point, the normal direction, *n*, was estimated as the local gradient of the binary mask. Along this direction, a one-dimensional intensity profile, $I\left(s\right)$, was extracted, where s denotes the position in millimeters relative to the boundary (s=0 on the contour, negative values inside the lesion, positive values outside).

The local contrast at a given boundary point was then defined as follows:

(10) $$C_{local}={max}_{s\in [-0.5,0.5]}I\left(s\right)-{min}_{s\in \left[-0.5,0.5\right]}I\left(s\right)$$

For each lesion, the distribution of local contrast values across all contour points was summarized by its mean and standard deviation:

(11) $$C=\frac{1}{M}\sum_{k=1}^{M}C_{local,k}{\;\sigma }_{C}=\sqrt{\frac{1}{M}\sum_{k=1}^{M}{\left(C_{local,k}-C\right)}^{2}}$$

where M is the number of sampled contour points.

Together, these parameters—contrast statistics within the margin band and local contrast along normal profiles—provide a robust, device-independent assessment of boundary sharpness. Increased sharpness typically indicates well-delineated lesions (more common in FTC and MTC), whereas blurred or heterogeneous boundaries suggest infiltrative growth, which is often associated with PTC [50,51].

- Boundary Blurring—Kullback–Leibler Divergence Relative to Normal Parenchyma

To quantitatively assess the distinctiveness of lesion boundaries, the Kullback–Leibler (KL) divergence was computed between the local intensity distribution at the lesion margin and the intensity distribution within normal thyroid parenchyma. This parameter was used as a measure of lesion-to-background separability—higher values indicate a well-defined boundary with clearly distinguishable signal properties, whereas lower values suggest boundary blurring and similarity to the surrounding tissue.

For each lesion, a margin of 1 mm thickness was generated around the lesion contour, as defined by the segmentation mask. Grayscale images were previously normalized relative to the mean echogenicity of normal thyroid tissue to ensure comparability of intensity distributions across cases. The intensity distribution was used to estimate a probability distribution for two regions: (1) the margin zone surrounding the lesion boundary and (2) a reference region within the adjacent healthy parenchyma.

The KL divergence ($D_{KL}$) was computed according to the following formula:

(12) $$D_{KL}\left(P∥Q\right)=\sum_{i=1}^{N}P\left(i\right)\cdot log\left(\frac{P\left(i\right)}{Q\left(i\right)}\right)$$

where *P*(*i*) denotes the probability of intensity in the *i*-th histogram bin within the margin region and *Q*(*i*) is the corresponding probability in the reference region.

Lower values of $D_{KL}$ were interpreted as indicative of poor boundary distinctiveness, with intensity distributions closely resembling those of the surrounding parenchyma. In contrast, higher values reflected greater divergence between lesion and background tissue, suggesting a sharp, well-delineated border. This measure served as an objective and observer-independent parameter for characterizing the degree of lesion separability and for supporting the differentiation of focal thyroid lesions.

#### 2.3.4. Structural Features

- Micro- and Macrocalcifications

To quantitatively assess the presence of calcifications in focal thyroid lesions, a detailed analysis of binary masks corresponding to calcified areas and total lesion area was performed. Potential calcifications were identified on ultrasound images by applying an intensity threshold: regions with normalized grayscale values exceeding 0.85 were considered highly echogenic and classified as potential calcifications. Subsequently, morphological filtering was used to eliminate small artifacts that did not meet predefined morphological criteria. The resulting binary masks were automatically generated and then verified by visual comparison with B-mode images to confirm the correct localization of hyper-echoic foci.

For each lesion, the total lesion area ($A_{lesion}$) and the area occupied by calcifications ($A_{calc}$) were calculated in square millimeters, based on the true spatial resolution of the image (pixel dimensions in the X and Y axes). In previous studies, the distinction between micro- and macrocalcifications was defined using both 1 mm and 2 mm thresholds. In this study, an intermediate value was adopted as a compromise between these criteria [52,53]. Calcifications were further classified as microcalcifications (≤1.5 mm) or macrocalcifications (>1.5 mm) according to their maximum transverse dimensions.

Two quantitative indicators were then computed: calcification density (for micro- or macrocalcifications), defined as the number of respective foci ($N_{micro}\;and\;N_{macro}$) per unit area of the lesion:

(13) $$D_{micro}=\frac{N_{micro}}{A_{lesion}}\;D_{macro}=\frac{N_{macro}}{A_{lesion}}$$

and calcification area ratio, defined as the percentage of the lesion area occupied by calcifications:

(14) $$R_{calc}=\frac{A_{calc}}{A_{lesion}}\times 100\%,$$

In addition, the spatial distribution of calcifications within the lesion was assessed. Based on the segmentation mask, the central region of the lesion was defined via morphological erosion of the full lesion mask, while the peripheral zone was defined as the difference between the original and eroded masks. This approach enabled differentiation between calcifications located near the lesion margin and those situated deeper within the lesion core. Such an analysis provided a more comprehensive, size-independent assessment of calcification patterns across focal thyroid lesions [52,53,54].

- Anechoic Areas (Cystic Components and Necrosis)

On B-mode ultrasound images, some focal thyroid lesions may contain anechoic regions, which appear as markedly hypoechoic areas within the tumor. The presence of such regions may indicate cystic components, liquefied necrosis, or fluid-filled zones within otherwise solid structures [55,56,57]. Therefore, one of the analyzed features was the presence and spatial characterization of anechoic areas within the lesion.

All ultrasound images were converted to grayscale and normalized relative to the mean intensity of normal thyroid parenchyma. Anechoic areas were defined as contiguous regions with intensity values below 0.2, located entirely within the lesion boundaries. To reduce the impact of noise and exclude isolated low-intensity pixels, morphological filtering was applied to retain only spatially coherent structures.

For each lesion, the total lesion area ($A_{lesion}$) and the area of anechoic regions ($A_{anechoic}$) were computed as described previously. The presence of anechoic areas was then quantified using the anechoic area ratio, defined as the percentage of the lesion area occupied by these regions:

(15) $$R_{anechoic}=\frac{A_{anechoic}}{A_{lesion}}\times 100\%$$

### 2.4. Statistical Analysis

All statistical analyses were performed using MATLAB R2023b (MathWorks, Natick, MA, USA). Prior to group comparisons, the distribution of continuous variables was assessed using the Shapiro–Wilk test, which is recommended as a sensitive method for evaluating normality in biomedical data [58]. Since most of the analyzed features did not meet the assumptions of normal distribution, the non-parametric Kruskal–Wallis test was used to compare the three histological subtypes: papillary (PTC), follicular (FTC), and medullary (MTC) thyroid carcinoma.

When significant differences were detected using the Kruskal–Wallis test, post hoc pairwise comparisons were performed using Dunn’s test, with significance level adjustment based on Šidák correction to control the risk of type I error associated with multiple comparisons [59,60].

A *p*-value < 0.05 was considered statistically significant. The analysis included all quantitative ultrasound-based features, including geometric parameters, echogenicity, texture metrics, boundary characteristics, presence of calcifications, and anechoic components. Statistical results are reported as *p*-values for features showing significant intergroup differences.

### 2.5. Multiparametric Classification Based on Quantitative Imaging Features

A three-class classification model based on the Random Forest algorithm (100 trees) was developed to differentiate between PTC, FTC, and MTC based on ultrasound-derived features. The dataset (244 images representing 122 lesions) was randomly divided into a training set (70%, n = 171) and a test set (30%, n = 73), with stratification to preserve class proportions. Each tree in the ensemble was trained on a bootstrap sample of the training set. At each node, a random subset of predictors (proportional to the square root of the total number of features) was considered for splitting. Splits were selected using the Gini impurity index, ensuring optimal separation of classes. Individual trees generated independent class predictions, and the final classification was determined by majority voting across all trees.

Model performance was evaluated using two strategies: (1) out-of-bag (OOB) validation, based on predictions from trees that did not include a given sample during training, and (2) an independent test set, providing an unbiased estimate of generalization performance [61,62,63,64].

After training the full model, feature importance was estimated using the OOB permutation method (OOBPermutedPredictorDeltaError). A feature was considered important if random permutation of its values increased classification error. Based on this ranking, the 10 most informative predictors were selected to construct a reduced model. The same training and validation procedures were applied to evaluate whether limiting the model to the most relevant features could preserve diagnostic performance while improving interpretability [65,66,67,68].

### 2.6. Software and Data Availability

All procedures for image analysis and feature extraction were implemented in MATLAB R2023b (MathWorks, Natick, MA, USA) using custom scripts. The processing pipeline included grayscale conversion, intensity normalization, segmentation refinement, quantitative feature computation, statistical analysis, and construction of a multiparametric classification model.

Anonymized B-mode ultrasound images and the corresponding segmentation masks used for quantitative analysis are available from the corresponding author upon reasonable request.

## 3. Results

### 3.1. Quantitative Evaluation of Single Ultrasound Features

#### 3.1.1. Morphological Feature Assessment: Shape and Complexity

The aspect ratio, describing the spatial orientation of the lesion, did not significantly differentiate thyroid cancer subtypes (*p* = 0.297). The lowest median values were observed in MTC (0.78), while the highest were noted in FTC (0.98), which may reflect the more regular, oval shape of follicular tumors. In PTC, the widest range of values was noted, including cases with an aspect ratio > 1, potentially indicating a more vertical, infiltrative growth pattern.

The perimeter-to-area ratio, reflecting boundary complexity, significantly differentiated tumor subtypes (Kruskal–Wallis test: *p* < 0.0001). Post hoc Dunn–Šidák analysis revealed significant differences between PTC vs. FTC (*p* < 0.0001) and MTC vs. FTC (*p* = 0.0002). The highest values were observed in the PTC group (median = 0.385), and the lowest in FTC (0.191), suggesting greater boundary irregularity in papillary carcinomas.

#### 3.1.2. Echogenicity and Intratumoral Texture Characteristics

Echogenicity showed significant differences among the three histological subtypes for both the mean (*p* = 0.0003) and median values (*p* = 0.0002). Post hoc analysis confirmed statistically significant differences for all pairwise comparisons: MTC vs. FTC (*p* ≤ 0.0002), MTC vs. PTC (*p* ≈ 0.014–0.016), and PTC vs. FTC (*p* ≈ 0.023–0.035). The lowest values were observed in MTC, and the highest in FTC. In contrast, the standard deviation of echogenicity did not significantly differ between groups (*p* = 0.1121).

Local entropy, representing the degree of signal disorder within the lesion, also showed significance (*p* = 0.0360), with a significant post hoc difference identified between MTC and FTC (*p* = 0.0486). Other texture features based on the gray-level co-occurrence matrix (GLCM) did not reach statistical significance, although contrast approached the significance threshold (*p* = 0.0565).

#### 3.1.3. Assessment of Tumor Margins

Intensity gradients and local contrast along the lesion boundary significantly differentiated between thyroid cancer subtypes. For the mean gradient, a significant difference was found between PTC and FTC (*p* = 0.0339), while the gradient standard deviation differed significantly between PTC and FTC (*p* = 0.0014) and between MTC and FTC (*p* = 0.0163). Similarly, mean profile intensity differed between PTC and FTC (*p* = 0.0430), and the standard deviation of profile intensities showed differences between PTC and FTC (*p* = 0.0009) as well as between MTC and FTC (*p* = 0.0180). The lack of significant differences between PTC and MTC for these features suggests that boundary-related parameters are most effective in distinguishing FTC from the other subtypes.

The Kullback–Leibler divergence, which quantifies the distinction between the lesion and the surrounding thyroid parenchyma, also showed significant differences (*p* = 0.0049), with post hoc analysis revealing a significant difference between MTC and PTC (*p* = 0.0165).

#### 3.1.4. Internal Composition and Calcification Patterns

Macrocalcifications and the percentage of the tumor area occupied by calcifications significantly differed across thyroid cancer subtypes (*p* = 0.0112 and *p* = 0.0435, respectively). Post hoc Dunn–Šidák analysis revealed significant differences between PTC and FTC (*p* ≈ 0.0081–0.0399). In contrast, microcalcifications did not significantly differ between groups (*p* = 0.7264).

Peripheral calcifications showed highly significant differences between subtypes (*p* < 0.00001). The highest median count was observed in FTC (17), markedly exceeding the values for PTC and MTC (median = 3). These differences were statistically significant for the FTC–PTC and FTC–MTC comparisons (*p* < 0.0001).

Anechoic (cystic) areas were most frequently observed in FTC lesions; however, the Kruskal–Wallis test did not show significant differences between groups (*p* = 0.7902), limiting the diagnostic utility of this feature as a standalone parameter.

### 3.2. Comparative Evaluation of Individual Quantitative Ultrasound Features

All analyzed ultrasound features are summarized in Table 2, which presents the statistical significance of differences between the three thyroid carcinoma subtypes (PTC, FTC, and MTC) for each pairwise comparison. Statistically significant differences are marked with an “X”. Based on this comparative analysis of individual imaging features, it can be observed that only certain parameters showed significant differences between selected group pairs. For instance, the mean and median echogenicity, as well as features describing boundary complexity, significantly differentiated PTC from FTC, as well as MTC from FTC. In contrast, other features—such as the presence of anechoic areas or selected GLCM-based texture metrics—did not independently show significant differences between the subtypes.

To reduce the impact of multicollinearity among features, a correlation analysis was performed for all extracted imaging parameters. Pearson correlation coefficients were calculated for each pair of features. Several pairs of variables exhibited strong linear correlations (|r| > 0.9), which may have led to redundancy and disproportionately influenced the classification model.

To minimize this redundancy, only one parameter was retained from each pair of highly correlated features. When selecting which variable to keep, priority was given to those that demonstrated statistical significance in the three-group analysis and offered greater clinical interpretability. Special emphasis was placed on features that could be more readily understood and applied in clinical practice.

This approach allowed the number of input variables to be reduced to 14 independent features, contributing to lower model variance and improved interpretability. The final set of features used for model construction is presented in Table 3.

### 3.3. Classification Model Based on Full Feature Set

The classification model based on the full set of 14 imaging features (Table 3) achieved an overall classification accuracy of 89.3%. However, class-wise performance varied in predicting specific tumor subtypes.

Class-wise performance, recalculated to match class sizes is summarized in Table 4 and revealed the following: for FTC, a precision of 75.0%, a recall of 64.3%, and an F1-score of 69.1%; for MTC, a precision of 88.2%, a recall of 83.3%, and an F1-score of 85.7%; and for PTC, a precision of 92.4%, a recall of 94.4%, and an F1-score of 93.4%. 

The confusion matrix presented in Table 5 shows that the most common misclassifications involved FTC being labeled as PTC and MTC being labeled as PTC, which may be attributed to overlapping morphological characteristics.

### 3.4. Feature Importance and Reduced Feature Model

Following the evaluation of the classification performance of the model based on the full set of features, an analysis of predictor importance was conducted. The permutation-based importance metric (OOBPermutedPredictorDeltaError) was used, which measures the increase in out-of-bag (OOB) error after randomly permuting the values of a given feature.

**Figure 1:**
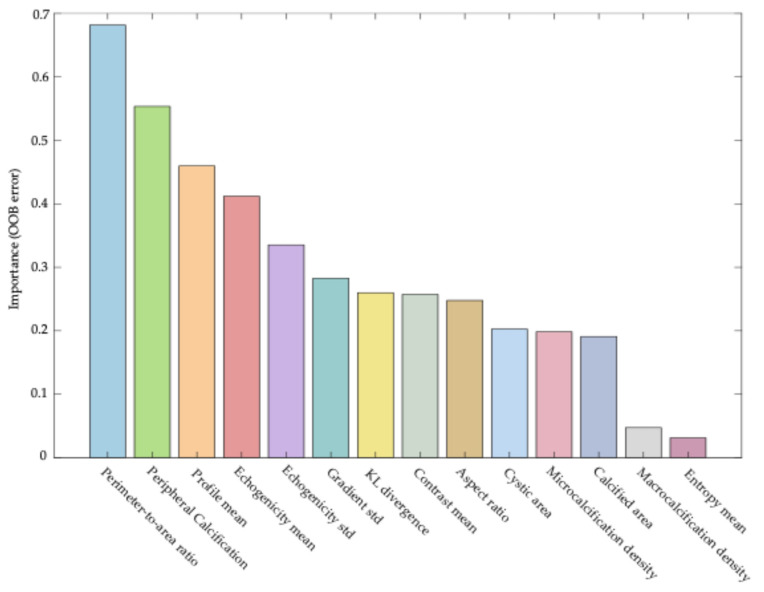
Importance of features in the Random Forest model. The vertical axis represents the increase in out-of-bag (OOB) classification error following random permutation of each feature, reflecting its relative contribution to model performance. Features are ranked in descending order of importance.

This analysis enabled ranking of the features according to their impact on the model’s predictive accuracy. High values of the permutation importance index indicated a significant contribution of the feature to classification decisions, whereas values close to zero or negative values suggested limited or no diagnostic relevance. The results of this analysis are presented as a bar plot in Figure 1, with features ordered in descending order of importance.

Among the features with the highest information value in the classification model were both morphological parameters, such as the perimeter-to-area ratio (feature #1), and features related to calcification, including the presence of peripheral calcifications (feature #2). Parameters associated with lesion echogenicity also played a significant role, including mean echogenicity (feature #4) and the mean boundary profile value (feature #3).

While some individual features demonstrated clear dominance in importance, the results highlight the value of a multidimensional approach, in which complementary information derived from different image aspects (morphology, texture, echogenicity, and calcifications) collectively contributes to accurate differentiation of thyroid cancer histological subtypes.

Based on these findings, the 10 most important predictors were selected to construct a simplified classification model. The aim of this step was to evaluate whether reducing the number of input variables could maintain high diagnostic accuracy while simplifying the model’s structure and improving interpretability.

The model based on the top 10 features achieved a classification accuracy of 91.8%, indicating strong predictive performance even with a reduced feature set. For the FTC class, the model reached a precision of 66.7% and a recall of 85.7%, resulting in an F1-score of 70.0%. In the case of MTC, the precision and recall were 70.0% and 77.8%, respectively, yielding an F1-score of 73.6%. For PTC, the model demonstrated very high recall (95.6%) and precision (96.1%), with an F1-score of 94.0%. Detailed results are presented in Table 6.

The confusion matrix presented in Table 7 shows that the most common misclassifications involved FTC cases being predicted as PTC (14.3%) and MTC cases being predicted as PTC (22.2%). This may be attributed to partial overlap in imaging features between these thyroid cancer subtypes.

A comparison of confusion matrices between the full model (based on 14 features) and the reduced model (using the 10 most important predictors) indicates comparable classification performance for both approaches. For the FTC class, the reduced model even achieved slightly higher classification accuracy (85.7% vs. 77.8% in the full model), suggesting that reducing the number of input variables did not impair recognition of this class. A slight decrease was observed for MTC, with classification accuracy decreasing from 83.3% to 77.8%, although misclassifications as PTC increased slightly (22.2% vs. 16.7%). For the PTC class, the full model showed slightly better performance (94.4% vs. 95.6%), but this difference had a negligible impact on overall classification accuracy.

These findings confirm that the simplified classification model, despite using fewer features, maintained high predictive performance—outperforming the full model in some cases. The slight decrease in accuracy for PTC was offset by improved recognition of FTC and MTC cases. Therefore, reducing the number of input variables may not only enhance the model’s interpretability but also increase its generalizability, particularly for underrepresented tumor subtypes.

## 4. Discussion

The results of the conducted analyses confirm that a quantitative approach to ultrasound assessment of thyroid nodules, based on objective and standardized B-mode parameters, enables effective differentiation of the three main histological subtypes of thyroid carcinoma: papillary (PTC), follicular (FTC), and medullary (MTC). The Random Forest model constructed using the full set of 14 imaging features achieved a high classification accuracy of 89.3%, which was preserved in the simplified version based on only the 10 most important predictors (91.8%). This confirms that a well-selected, reduced set of features can provide equally high diagnostic performance while improving model interpretability and reducing the risk of overfitting.

In the permutation-based importance analysis, four predictors were identified as having the highest diagnostic relevance, each representing a distinct category of quantitative ultrasound features: perimeter-to-area ratio (morphology), peripheral calcification (structural features), profile mean (boundary sharpness), and echogenicity mean (internal echogenicity). Each of these features reflects a different aspect of the nodule’s sonographic appearance, namely, irregular margins, the presence of peripheral calcifications, contrast along the margin, and internal texture, respectively. This complementary integration of multiple imaging domains contributed to the high classification performance and enhanced the interpretability of the model in relation to the underlying histopathological differences among thyroid cancer subtypes.

In this discussion, only selected features from the broader set of evaluated parameters are highlighted, as detailed elaboration on all of them would exceed the scope of this article.

Among the listed predictors, perimeter-to-area ratio demonstrated the highest predictive importance score (0.70), indicating its key role in the classification process. Its diagnostic value stems from its biological interpretability. Papillary thyroid carcinoma (PTC) typically exhibits infiltrative growth and papillary architecture, resulting in irregular, spiculated margins and elevated perimeter-to-area ratios [69,70,71]. In contrast, follicular thyroid carcinoma (FTC) more often presents as encapsulated lesions with smooth margins, leading to lower perimeter-to-area values [70,72,73]. Medullary thyroid carcinoma (MTC) shows a more variable morphology but often also smooth margins [70,74].

Regardless of these biological differences, the effectiveness of this approach relies not only on the choice of the metric itself but also on the methodology used to extract it. The accuracy of margin analysis is particularly sensitive to the method of segmentation, especially in the case of nodules with ill-defined or infiltrative borders. Unlike fully automated approaches, which frequently struggle with precise segmentation in low-contrast areas, semi-automated and iterative techniques allow for more reliable and reproducible results. Recent studies have demonstrated that integrating methods such as active contours, morphological filtering, and specialized boundary-sensitive modules significantly improves segmentation performance, especially in challenging cases [75,76,77,78,79]. The combination of boundary-based features with morphological operations such as dilation and erosion enables more accurate contour delineation, even in the presence of blurred edges [75,77,79].

Importantly, unlike subjective features such as “irregular margins” assessed by radiologists, the perimeter-to-area ratio is an entirely objective and reproducible metric. This makes it particularly attractive in the context of malignancy risk stratification and automation. It may assist clinicians in biopsy decision-making and serve as a core component of future AI-based decision support systems.

Due to its biological relevance and interpretability, the perimeter-to-area ratio can also serve as a conceptual bridge between traditional image-based diagnostics and modern machine learning algorithms. Incorporating this feature into predictive models enhances both diagnostic accuracy and the transparency of the decision-making process, which is essential for clinical acceptance of AI-assisted diagnostic tools.

Peripheral or rim calcifications have a distinct diagnostic value, as their quantitative assessment, such as the number of high-intensity pixels along the lesion contour, reflects the presence of calcifications at the tumor border. In the TI-RADS and EU-TIRADS classifications, it is emphasized that peripheral calcifications, particularly those with an “eggshell” appearance and interrupted continuity, may be associated with an increased risk of malignancy, whereas regular, continuous rim calcifications are typically found in benign lesions [9,10,11,80,81,82]. Studies have shown that central microcalcifications are most commonly observed in papillary thyroid carcinoma, while peripheral macrocalcifications may occur in papillary, follicular, and medullary thyroid carcinomas, with their prognostic significance depending on morphology and continuity [80,82,83,84]. Interrupted rim calcifications are associated with a higher risk of malignancy and may indicate an infiltrative growth pattern, whereas continuous “eggshell”-type calcifications are characteristic of benign lesions [80,81,82].

Statistical analyses confirm that the presence of peripheral calcifications—particularly when combined with other suspicious features such as solid composition or irregular margins—is associated with an increased risk of malignancy [81,82]. However, the presence of macrocalcifications or peripheral calcifications alone, in the absence of other suspicious characteristics, does not necessarily indicate a high risk of cancer [85,86,87]. Recent studies suggest that automated, quantitative analysis of calcifications—including their number, distribution, intensity, and distance from the lesion margin—may aid in differentiating benign from malignant nodules and in predicting metastatic risk, especially in papillary thyroid carcinoma [81,85,88]. Deep learning-based models allow for automatic detection and assessment of calcifications, and the parameters obtained show strong agreement with expert radiologist evaluation and significant prognostic value, although their use in routine clinical practice remains limited. The integration of such tools with AI-based diagnostic systems has the potential to substantially improve the classification of lesions with ambiguous echogenicity or architecture, thereby supporting clinical decision-making [85,88].

The parameter profile mean, which describes the average contrast at the boundary of a lesion relative to the surrounding thyroid parenchyma, plays a key role in differentiating nodules based on the architecture of their margins. A high value of this metric reflects a sharp, well-defined transition between the lesion and adjacent tissue, corresponding to the “sharp margins” criterion in the TI-RADS and EU-TIRADS classifications, where indistinct or irregular borders are associated with a higher risk of malignancy [9,10,11]. Numerous studies have confirmed that irregular, poorly defined margins are characteristic of infiltrative lesions such as papillary or medullary thyroid carcinoma, whereas benign lesions and certain subtypes of follicular carcinoma typically exhibit smooth, encapsulated contours [89,90,91]. Intensity profile analysis at the interface between parenchyma and lesion enables a quantitative assessment of these features, which may provide valuable insights into the growth pattern and malignant potential of the nodule [90,92,93]. Automated computation of the profile mean based on averaged intensity profiles around the lesion contour ensures high reproducibility and eliminates subjective variability, thereby supporting standardization and integration with artificial intelligence-based diagnostic tools [92,94].

In the presented study, the lowest values of echogenicity were observed in medullary thyroid carcinoma (MTC), which aligns with findings from previous reports [13,95]. For follicular thyroid carcinoma (FTC), the literature suggests a generally higher echogenicity, although clear comparative data remain limited [96,97]. Hypoechogenicity of a thyroid nodule is a well-established indicator of malignancy risk, particularly in papillary thyroid carcinoma (PTC), as demonstrated by both ultrasound and histopathological studies [13,96,97]. Ultrasonographic studies have shown that PTC is most commonly hypoechoic, while MTC may exhibit either low or mixed echogenicity. FTC, along with other follicular-patterned lesions, more frequently demonstrates echogenicity similar to that of the surrounding thyroid or only mildly reduced [13,95,96,97].

In the presented classification model, the echogenicity mean was computed using a fully automated method that included local normalization relative to the background tissue. This approach enhanced the robustness and reproducibility of the measurement, reducing the influence of inter-device variability [98].

The results further support the effectiveness of multiparametric models in differentiating thyroid focal lesions. Consistent with the literature, combining morphological, textural, and intensity-based features improves both sensitivity and specificity, while also increasing model resilience to noise and variability across different ultrasound systems [99,100,101,102,103]. Previous studies have reported high predictive performance for such hybrid models—for instance, Random Forest classifiers achieving accuracies of up to 96.1% [100] and other advanced algorithms exceeding 95% [100,103,104]. An important advantage of such models is the low correlation among key predictors, which limits redundancy and enhances classification efficiency [100,102]. Moreover, these models have demonstrated strong performance both with the full feature set and after dimensionality reduction, confirming their practical applicability in clinical diagnostics.

Recent advances in deep learning have introduced novel architectures for image analysis, including attention-based multiview frameworks [105], style-contrastive networks for content–style disentanglement [106], and transformer-based weakly supervised segmentation with adversarial training [107]. While these methods achieve state-of-the-art performance in various computer vision domains, their application in endocrine oncology remains limited, primarily due to the lack of clinical interpretability and the challenges in validating such black-box models. In contrast, the quantitative ultrasound features employed in our study are transparent, biologically interpretable, and directly linked to histopathological correlates, thereby supporting reproducibility and clinical trust.

Unlike deep learning methods, which are often perceived as opaque “black-box” systems, the approach presented here offers complete transparency in both analytical processing and interpretation of results. Each feature was designed based on a biologically grounded rationale, allowing for clinical interpretation of the measured values and supporting transparency in the decision-making process.

It is also worth noting that much of the existing literature has focused on binary classification tasks—distinguishing benign from malignant lesions—without addressing finer distinctions among thyroid cancer subtypes [108,109,110]. In contrast, the present study tackled a three-class classification problem involving PTC, FTC, and MTC subtypes that differ not only in prognosis but also in architectural patterns, morphology, and biological behavior. Therefore, the proposed model aligns with current trends in quantitative ultrasound analysis while extending their application to more complex and clinically meaningful classification tasks.

Despite the high classification performance and the use of a fully quantitative and transparent approach, this study has several limitations. First, the analysis was conducted retrospectively and was based on data from a single institution, which may limit the generalizability of the findings across more diverse patient populations. Second, although the segmentation method was optimized and semi-automated, it still required expert supervision, which could affect reproducibility in settings with varying levels of operator experience. Third, the classification model was developed using B-mode ultrasound images acquired in two orthogonal planes (transverse and longitudinal). While this enabled the extraction of key morphological and structural features, three-dimensional ultrasound data were not included, which might have provided a more comprehensive representation of tumor architecture—particularly with respect to irregular margins, spatial distribution of calcifications, and internal heterogeneity.

In addition, the robustness of the proposed method to variations in ultrasound equipment, acquisition protocols, and device manufacturers has not yet been validated. Although Random Forest classifiers are relatively computationally efficient, the complete pipeline—including preprocessing and semi-automated segmentation—may impose additional computational demands, which could limit practical deployment in resource-constrained healthcare environments.

Future research should therefore address these challenges by including larger and more diverse multi-center cohorts, validating the approach across different ultrasound platforms, incorporating three-dimensional data, and optimizing computational efficiency. Such efforts will be crucial to ensure the broad applicability and real-world feasibility of quantitative ultrasound as a clinically trusted decision-support tool.

## 5. Conclusions

The application of quantitative ultrasound, supported by computational methods and machine learning, enables effective differentiation of the three major histological subtypes of thyroid cancer: papillary (PTC), follicular (FTC), and medullary (MTC). The Random Forest model, built upon selected morphological, structural, and textural features, achieved high classification accuracy (91.5%) while maintaining transparency and clinical interpretability.

The high sensitivity observed for PTC reflects the model’s strong performance in detecting the most common thyroid cancer subtype, which is of substantial clinical relevance. At the same time, the high precision in classifying FTC and MTC suggests a low rate of misclassification for these less common but potentially more aggressive subtypes. The limited overlap in misclassifications between FTC and MTC, together with the model’s robust diagnostic performance, highlights the potential of this approach as a clinically useful decision-support tool.

Validation of these findings requires further studies involving larger, more diverse cohorts and multicenter external validation to assess model performance in real-world settings. Integrating semi-automated segmentation and classification methods into the diagnostic workflow for thyroid nodules could reduce the number of unnecessary biopsies, support the implementation of personalized medicine strategies, and improve overall patient care efficiency.

## Figures and Tables

**Table 1:** Most commonly reported ultrasound features of the three main thyroid cancer subtypes.

Ultrasound Feature	Papillary Thyroid Carcinoma (PTC)	Follicular Thyroid Carcinoma (FTC)	Medullary Thyroid Carcinoma (MTC)
Echogenicity	Hypoechoic, sometimes heterogeneous [19,20,21,22]	Iso- or hypoechoic [18,20,21,22]	Hypoechoic [13,19,23]
Margins	Irregular, ill-defined [19,20,21,22]	Regular or irregular if invasive [18,20,21,22]	Smooth, well-defined (sometimes ill-defined) [13,19,23]
Calcifications	Microcalcifications (psammoma bodies) [19,20,21]	Micro- and macrocalcifications, often peripheral (“eggshell”) [18,20,21]	Micro- and macrocalcifications (amyloid deposits, shadowing) [13,19,23]
Internal structure	Solid, possibly heterogeneous [19,20,21,22]	Solid, heterogeneous [18,20,21]	Solid, possibly homogeneous [13,19]
Shape (aspect ratio)	“Taller-than-wide” (common) [18,19,20,21]	Oval or irregular [18,20,21]	Variable, round or oval [13,19]
Vascularity (Doppler)	Often increased, chaotic internal pattern [18,19]	Moderate, mixed pattern [17,18]	Increased, central and peripheral [13,17,19]
Elastography	High stiffness [19]	Variable, often intermediate [18]	High stiffness [18,23]
Presence of capsule	Absent or interrupted capsule [18,21]	Often infiltrated, extracapsular extension [18,21]	Absent [18]
Cystic component	Rare, usually <10% of volume [18,19]	Rare [18]	Often present, especially in larger lesions [13]
Lymph node metastases	Common at diagnosis [18,19]	Less common [13,19]	Common [13,19]

**Table 2:** Results of a two-step statistical analysis. First, the Kruskal–Wallis test was applied to assess overall differences in the distribution of quantitative ultrasound features among thyroid cancer subtypes. Reported *p*-values in this column indicate the global significance level for each parameter. For features with statistically significant results (*p* < 0.05), post hoc Dunn–Šidák pairwise comparisons were performed to identify specific group differences, while for non-significant parameters post hoc comparisons were not applicable (marked as “–”). Statistically significant *p*-values are shown in bold.

Parameter Group	Parameter	Kruskal–Wallis (*p*)	Post Hoc Dunn–Šidák Comparisons (*p*)		
			PTC vs. FTC	PTC vs. MTC	MTC vs. FTC
Morphological Features	Aspect ratio	0.297	–	–	–
	Perimeter-to-area ratio	**<0.0001**	**<0.0001**	0.0674	**0.0002**
Internal Architecture	Echogenicity (mean)	**0.0003**	**0.0234**	**0.0141**	**0.0002**
	Echogenicity (median)	**0.0002**	**0.0352**	**0.0162**	**0.0019**
	Echogenicity (std)	0.1121	–	–	–
	Local entropy (mean)	**0.0360**	0.2147	0.1863	**0.0486**
	Local entropy (std)	0.0673	–	–	–
	Contrast (mean)	0.0565	–	–	–
	Correlation (mean)	0.1569	–	–	–
	Homogeneity (mean)	0.9859	–	–	–
	Energy (mean)	0.7586	–	–	–
Margin Assessment	Gradient (mean)	**0.0400**	**0.0339**	0.9464	0.2416
	Gradient (std)	**0.0021**	**0.0014**	0.8999	**0.0163**
	Profile (mean)	**0.0014**	**0.0430**	0.8193	0.3867
	Profile (std)	0.0443	**0.0009**	0.9909	**0.0180**
	KL divergence	**0.0049**	0.1268	**0.0165**	0.6894
Structural Features	Microcalcification density	0.7264	–	–	–
	Macrocalcification density	**0.0112**	**0.0081**	0.9811	0.0834
	Calcified area %	**0.0435**	**0.0399**	0.9989	0.1074
	Peripheral calcification	**<0.0001**	**<0.0001**	**0.0127**	**<0.0001**
	Cystic area %	0.7902	–	–	–

**Table 3:** Final set of imaging features after collinearity reduction—results of the Kruskal–Wallis test with Dunn–Šidák post hoc comparisons for parameters differentiating thyroid cancer subtypes (PTC, FTC, and MTC). Exact *p*-values are provided; statistically significant differences (*p* < 0.05) are shown in bold.

Parameter Group	Parameter	Kruskal–Wallis (*p*)	Post Hoc Dunn–Šidák Comparisons (*p*)		
			PTC vs. FTC	PTC vs. MTC	MTC vs. FTC
Morphological Features	Aspect ratio	0.297	–	–	–
	Perimeter-to-area ratio	**<0.0001**	**<0.0001**	0.0674	**0.0002**
Internal Architecture	Echogenicity (mean)	**0.0003**	**0.0234**	**0.0141**	**0.0002**
	Echogenicity (std)	0.1121	–	–	–
	Local entropy (mean)	**0.0360**	0.2147	0.1863	**0.0486**
	Contrast (mean)	0.0565	–	–	–
Margin Assessment	Gradient (std)	**0.0021**	**0.0014**	0.8999	**0.0163**
	Profile (mean)	**0.0014**	**0.0430**	0.8193	0.3867
	KL divergence	**0.0049**	0.1268	**0.0165**	0.6894
Structural Features	Microcalcification density	0.7264	–	–	–
	Macrocalcification density	**0.0112**	**0.0081**	0.9811	0.0834
	Calcified area %	**0.0435**	**0.0399**	0.9989	0.1074
	Peripheral calcification	**<0.0001**	**<0.0001**	**0.0127**	**<0.0001**
	Cystic area %	0.7902	–	–	–

**Table 4:** Classification performance metrics for the model built using the full feature set.

Class	Precision (%)	Recall (%)	F1-Score (%)
FTC	75.0	64.3	69.1
MTC	88.2	83.3	85.7
PTC	92.4	94.4	93.4

**Table 5:** The confusion matrix for the model built using the full feature set.

True Class/ Predicted Class	FTC (pred)	MTC (pred)	PTC (pred)
FTC (true)	64.3	0.0	35.7
MTC (true)	0.0	83.3	16.7
PTC (true)	3.3	2.2	94.4

**Table 6:** Classification performance metrics for the model built using the 10 most informative features.

Class	Precision (%)	Recall (%)	F1-Score (%)
FTC	66.7	85.7	75.0
MTC	70.0	77.8	73.6
PTC	96.6	95.6	96.1

**Table 7:** Confusion matrix (%)—classification based on the 10 most important features.

True Class/ Predicted Class	FTC (pred)	MTC (pred)	PTC (pred)
FTC (true)	85.7	0.0	14.3
MTC (true)	0.0	77.8	22.2
PTC (true)	1.1	3.3	95.6

## Data Availability

Anonymized B-mode ultrasound images and the corresponding segmentation masks used for quantitative analysis are available from the corresponding author upon reasonable request.
